# Repeated systemic inflammation was associated with cognitive deficits in older Britons

**DOI:** 10.1016/j.dadm.2015.11.009

**Published:** 2015-12-28

**Authors:** Gindo Tampubolon

**Affiliations:** University of Manchester, Manchester, UK

**Keywords:** C-reactive protein, Fibrinogen, Episodic memory, England

## Abstract

**Introduction:**

The relationship of C-reactive protein (CRP) to cognition in the older old group (≥75 years) has recently been found positive on both sides of the Atlantic. We hypothesized that higher levels of CRP and fibrinogen are related to worse episodic memory throughout later life (≥50 years).

**Methods:**

Data are drawn from older Britons free of dementias in the English Longitudinal Study of Aging 2004–2013. We applied growth trajectory models to repeated observations of episodic memory, CRP, and fibrinogen levels (and sociodemographic confounders). We accounted for practice effects in repeated tests of cognition.

**Results:**

Higher levels of both inflammatory markers were associated with worse episodic memory, where a fibrinogen effect is evident throughout later life (coefficient −0.154; 95% confidence interval [CI] −0.254 to −0.054). Most importantly, the CRP effect is strongly negative among the older old group (coefficient −0.179; CI −0.320 to −0.038).

**Discussion:**

Higher levels of fibrinogen are detrimental to older people's cognition, and among the older old, raised CRP levels are comparably deleterious. Repeated measures of inflammation can be considered in clinical practice as part of a response to the challenge of dementias.

## 1 Introduction

Dementias have a long period of preclinical development [1], [2]. With increase in life expectancy of the aging population, it is all the more important to understand early changes in cognitive function that presage cognitive impairment. Systemic inflammatory markers have been investigated for this purpose in middle aged and older people and have been found to be associated with many cognitive abilities including episodic memory, executive function, and global cognition [3], [4], [5], [6], [7], [8], [9], [10], [11], [12].

Two biomarkers are often used in studies of cognition and inflammation: fibrinogen and C-reactive protein (CRP). Epidemiologic, genetic, or pharmacologic studies on the relationship of fibrinogen to cognition suggest that higher levels of fibrinogen in the peripheral system are related to worse cognition [13], [14], [15], [16]. In the Aspirin for Asymptomatic Atherosclerosis Trial of older people in Scotland (aged 50–80 years), higher levels of baseline fibrinogen and CRP were associated with worse cognition during follow-up in cross-sectional design [9]. Also in Scotland, the Edinburgh Artery Study (aged 55–74 years) found that higher levels of fibrinogen at baseline predicted cognition deficits 14 years later in cross-sectional design [8]. The baseline levels were also associated with cognitive decline in change-scores longitudinal design, where only cognition is repeatedly measured.

Epidemiologic studies of the link between CRP and cognition have found results that cover the negative to positive spectrum of associations. In the Rotterdam Study (aged 55–106 years), higher levels of CRP at baseline were associated with worse cognition in cross-sectional design, but the baseline CRP levels were not associated with decline in cognitive function in change-scores longitudinal design [11]. Across the Atlantic, the Health, Aging, and Body Composition study found that being in the highest tertile of CRP levels at baseline had the same odds as being in the lower tertiles when predicting 4 years cognitive decline in change-scores longitudinal design [4]. Weak evidence is also found in the British Whitehall II study (mean age 48, standard deviation [SD] 6 years) where levels of CRP at baseline were associated with worse reasoning and vocabulary functions in cross-sectional design but were not associated with cognitive decline in change-scores longitudinal design [6]. Remarkably in the older old (age ≥75 years), raised CRP is associated with better cognition [10], [17], suggesting some genetic factors are responsible; see also [2]. An active inflammatory system may be found protective, delaying cognitive decline among the older old.

From a clinical perspective, this literature on peripheral inflammation has yet to bear fruit. Some are equivocal about the possibility [16], but others are less sanguine about the use of blood-based biomarkers in practice [11]. Such a view may be hasty given the weaknesses in the literature. First, it is unclear whether the positive relationship between inflammation and cognition applies to the younger old (50–75 years), a group of some importance given the well-known long duration of development of severe cognitive impairment. Second, many of these studies used one baseline measure of inflammation against repeated measures of cognition, effectively explaining change scores or decline as the dependent variable (change-scores longitudinal design). This poses two problems, one of which applies to any outcome (dilution bias), whereas the another applies specifically to cognition (practice effect). As is widely acknowledged in longitudinal studies, such change-scores design is susceptible to regression dilution bias [2], [3], [4], [5], [6], [7], [8], [11], [18]. It is possible that prospectively collected repeated measures of both cognition and biomarkers will modify the results mentioned.

In addition, a well-known threat to inference in longitudinal studies of cognition arises from practice effects [19], [20], [21], [22]. Apparent change in the test scores at two time points is partly down to the fact that participants have completed the same tests before. In the pages of this journal, it has been suggested that practice effects are “large, pervasive and under-appreciated” [22]. So in change-scores longitudinal design, if dilution bias can compromise analysis of any health outcomes, including physical and cognitive functions, practice effects can additionally compromise cognitive outcomes. Together, they render many biomarkers studies of cognitive decline doubly unsafe and unsatisfactory.

Finally, cognition in older people is maintained through mechanisms involving various biological and social factors. Careful accounting of cognitive deficits in later life requires social factors to be considered as well.

To address these weaknesses, this study used the English Longitudinal Study of Aging (ELSA) 2004–2013, a nationally representative prospective longitudinal study of older people (≥50 years) with repeated measures of the exposures, the outcomes and other demographic, economic, chronic conditions, and health behaviors as well as social factors. This study aimed to test two hypotheses. First, levels of high sensitivity CRP are related to worse episodic memory in younger old (<75 years) and in older old (≥75 years) Britons. Second, analogously, levels of circulating fibrinogen are independently related to worse episodic memory.

This measure of cognition is an important and popular measure in research on cognitive aging since it is easy to collect, easy to understand, and at the same time has been shown to be instrumental in life changing decisions such as pension decisions [23], [24], [25]. So not only does it enable research replication or cross-country comparison, it is also immediately relevant.

## 2 Materials and methods

### 2.1 English Longitudinal Study of Aging

The ELSA is the primary resource for a nationally representative aging study of the English older population; it was started in 2002 and subsequent waves follow biennially. In every even-numbered wave, a nurse assessment is given to collect biomedical information. The repeated biomarkers and episodic memory variables are, therefore, available from 2004 to 2005, 2008 to 2009, and 2012 to 2013 waves. The data are freely available from the UK Data Archive (www.data-archive.ac.uk) as study number 5050. More details of the study are given elsewhere [26], [27], [28], [29], [30], [31].

### 2.2 Ethics

Ethical approval for all the ELSA waves was granted from the National Research and Ethics Committee of the UK National Health Service www.nres.nhs.uk. The University of Manchester's institutional review board has exempted this study because it used publicly available anonymized secondary data.

### 2.3 Dependent variable

The dependent variable is episodic memory, the sum of delayed and immediate recall, available in all waves [23]. It is notable that this variable is also available in its sister study, the US Health and Retirement Study [32].

### 2.4 Independent variables

Information from respondents aged 50 to 89 years was used because age is capped at 90 years in ELSA. Demographic covariates include sex, age and squared-age to capture possible curvilinear trajectories [20], [21], [33]. To capture practice effects, an indicator variable is constructed, coding 1 if a respondent has had an episodic memory test in any previous wave, 0 otherwise; following others [21], [34].

Blood samples were collected in three waves and kept deep-frozen until analysis at the Newcastle NHS hospital laboratory. Plasma samples were analyzed for fibrinogen using an ACL TOP CTS analyzer. The change in light transmission caused by the conversion of soluble fibrinogen in plasma to cross-linked insoluble fibrin is monitored by the analyzer, and the clotting time threshold is determined to be 37% of the total change. The clotting time is directly related to the concentration of fibrinogen in the plasma and this time is converted to concentration in g/L. Plasma samples were also analyzed for high sensitivity CRP, applying a particle-enhanced immunoturbidimetric assay, using Roche Modular P analyzer. CRP in the sample reacts specifically with anti-human CRP antibodies coated on the latex particles to yield insoluble aggregates. The turbidimetric absorbance of these aggregates is proportional to the CRP concentration in mg/L in the sample. Because CRP distribution is skewed, its log transform is used.

Cognitive functions are known to be affected by physical function problems. All three dimensions of physical function are used in a total scores of physical function problems, including (instrumental) activities of daily living, mobility, and muscle functions [27]. Like other health functions, cognitive functions are also shaped by social determinants of health [26], [35], [36]. They include threefold social class (managerial, intermediate, and routine-manual as reference), wealth tertiles (top, middle, and bottom as reference), marital status (married/cohabiting and other as reference), education (some college and high school or less as reference), and ethnicity (minority and Caucasian white as reference). Social contact is entered as an indicator of making contact with friends once a week or more by email, by phone, in writing, or in person.

Based on positive medical history (self-report of “has been diagnosed by professionals”), a series of indicators about chronic conditions are also included, covering diabetes, cancer, hypertension, lung disease, heart condition, and stroke. Depression score is elicited using the Center for Epidemiologic Studies Depression scale (eight items) and is entered as a continuous variable.

Behavioral risk factors known to be effective in cross-sectional studies include smoking (current smoker and not current smoker as reference), drinking (days in a week having a drink), and physical exercise (rigorous, moderate physical exercise and mild or less as reference) [24].

### 2.5 Statistical analysis

Following theoretical [33] and empirical [20], [37] accounts of cognitive aging in Britain, a quadratic trajectory model is applied with random intercepts and random age slopes. This model, also known as a random-effects model or growth curve model, accounts for variations within individuals and across individuals. This model also gives consistent estimates of the effects of biomarkers when some participants leave the study, assuming missingness at random. This assumption means that, conditional on all the extensive covariates being accounted for, the differences between the dropouts and the stayers are due to mere chance [20]. We built a sequence of two models with increasing completeness addressing the gaps mentioned. First, the inflammations model included (in addition to the extensive confounders) the two key exposures of fibrinogen and log CRP levels. Second, to check whether among the older old the associations were reversed, the interactions model had two additional factors (an interaction between age ≥75 years and fibrinogen levels; analogously with age ≥75 years and log-CRP levels). This cutoff was chosen following the literature [10], [17]. All analyses are done in Stata 13 (StataCorp LP, College Station, TX, USA).

## 3 Results

The analytic sample is made up of those with complete covariates (Table 1) and has 54.4% females and 45.6% males with age ranging from 52 to 89 (mean = 67.3 years, SD = 8.4 years). Education can obscure age-related change in cognition due to education conferring test taking abilities, and this sample has 31.3% educated to college levels. Social inequalities in health are controlled for by also including wealth and occupational social class. This sample has 38.1% professionals or managers and about the same fraction held routine manual occupations. On average, the sample member reported nearly two physical problems (SD = 2.8). In this description, depression score has been grouped into two: none to 3 (88%) and 4 or more as depressed or “case-ness” (12%). On health behaviors of the sample, nearly four in five engaged in moderate physical exercise at least once a week and nearly one in three reported vigorous physical exercise during the same period.

The results from the sequence of models explaining episodic memory are given in Table 2 (coefficients and 95% confidence intervals [CI] in parentheses). In all models, episodic memory follows a nonlinear age trajectory [20], [21], [37]. (The complete coefficients and CIs are given in the Supplementary Material.) The baseline model shows that higher levels of fibrinogen are associated with worse episodic memory. The magnitudes and signs of fibrinogen associations remain similar along the sequence of models. Thus, in the interaction model, fibrinogen has a coefficient of −0.154 and CI of −0.254 to −0.054. The interaction model also shows that higher levels of CRP are associated with worse episodic memory among the older old (coefficient −0.179; CI −0.320 to −0.038). So both fibrinogen and CRP levels are associated with lower levels of episodic memory, particularly among the older old.

## 4 Discussion and conclusion

Severe cognitive impairment is known to be preceded by a long period of preclinical development [1], [38]; and the role of repeated inflammation in this development is examined here. In a nationally representative prospective longitudinal study, both high sensitivity CRP and fibrinogen levels were associated with cognitive deficits. Although the CRP effect was observable among the older old (≥75 years), the fibrinogen effect was observable earlier from the middle age (≥50 years).

Cognitive function, like other health outcomes [35], [36], is found to show strong social patterning along markers of socioeconomic positions of wealth, occupation, and education. In particular, the middle and top tertiles of wealth distribution showed significantly higher levels of cognitive function in later life (≥50 years), in fact showing a monotone increase. A gradient is also presented with respect to education; compared with the routine-manual class, those with intermediate occupations showed higher levels of cognitive function and those with managerial or professional occupations showed still higher levels of cognitive function.

The study has some limitations. On the choice of outcome, episodic memory is only one among many cognitive abilities that change during cognitive aging. Different cognitive abilities have shown different patterns of change over time [23], [39]. In parallel, different parts of the brain are activated in carrying out different cognitive tasks. Episodic memory, however, is important for many day-to-day and momentous decisions in later life, such as choosing retirement provision [25]. On the main exposure, inflammation is a complex biological process as it is simultaneously protective and destructive. The use of only two inflammatory markers is clearly limited, although the set examined here offers the advantage of examining inflammation and hemostasis. The role of the *APOE* gene is increasingly clear in the pathogenesis of dementias, but this information is unfortunately lacking for all respondents in this sample. This study directly entered an indicator of practice instead of a random effect of practice [21], [34] due to the small variation over the limited (three) waves; with more future waves, a random effect of practice can be used instead. Finally, dropouts from longitudinal study of aging can compromise inferences. This study responded to this by applying random-effects models estimated using maximum likelihood, which are known to give consistent estimates under missing at random assumption [21].

A unique advantage of the materials used here arises from its longitudinal design with repeated measures not only of episodic memory but also of high sensitivity CRP and fibrinogen. Not least, repeated measures of other risk factors and confounders were also part of the design. Another feature of the design is the nationally representative nature of ELSA, which goes someway to ensuring that the patterns of associations at work in the population can be extracted without being unduly influenced by specific patterns in some volunteer or selected samples.

These findings were in contrast to many conclusions in the literature. Unlike a few studies on limited samples which found elevated CRP to be protective, our data showed this to be potentially detrimental in the national sample. If high CRP levels are harmful for the older old, raised fibrinogen levels are harmful for both younger and older old (before and after 75 years). This longer pattern of fibrinogen association tallies with results in the animal and pharmacologic studies which elucidate the role of fibrinogen in both compromising the blood-brain barrier (BBB) and aiding inflammatory cascade [13], [14].

On mechanisms involving fibrinogen and CRP in the accumulation of cognitive deficits, much can be understood from dementias pathology. We are not, however, suggesting that cognitive deficits present in the data are likely to lead to severe cognitive impairment or dementias for these groups. Fibrinogen is the inactive precursor of fibrin, the primary protein component of a blood clot. Fibrinogen, a large complex molecule, is excluded from the brain by the BBB, as part of the normal functioning of the BBB, which also includes tight controls of communication between the central nervous system and the circulation. BBB damage has been found in mice with Alzheimer's disease (AD) brain and in patients with AD [13]. Through the increasingly permeable BBB, fibrinogen gains access to the brain and accumulates in the extravascular space, inducing pathology in the nervous systems [14], [15].

The BBB membrane structure is made up of special endothelial cells; it functions to protect the central nervous system from the peripheral system. This function can be weakened when tight junctions between endothelial cells are changed due to inflammation resulting in increased BBB permeability, allowing larger molecules such as fibrinogen to pass through the structure. Genetic and pharmacologic studies have furnished evidence supporting this fibrinogen and inflammation hypothesis [38].

In addition to extravascular disturbance to the brain, cerebrovascular dysfunction was also presented in AD brains, including cerebral amyloid angiopathy where amyloid-β (Aβ) is deposited as plaques in the blood vessels. Fibrinogen bound to Aβ is not efficiently cleared from the brain because hemostasis is altered in AD [14]. Because the brain needs a smooth supply of oxygen and glucose, as it lacks long-term energy stores, the plaques damage the cerebrovascular structure and compromise its function, thereby inducing cerebral hypoperfusion. The levels of cerebral hypoperfusion and the degree of dementia have been reported [13].

Aβ deposits on the walls of the blood vessels in cerebral amyloid angiopathy not only disturb the blood flow but also degenerate the cells of the walls and promote further inflammation. Thus, the deposition of Aβ in both extravascular and cerebrovascular space contribute to the neuroinflammation cascade [15], [38].

Finally, peripheral immune signals can be transmitted via the autonomic system to microglial cells in the brain. In turn, chronic microglial activation and upregulated inflammatory processes converge in the pathway of tau hyperphosphorylation [40]. This escalates further the neuroinflammatory process and neuronal death, ending in irreversible tissue damage [41]. The multiple processes involving hemostasis and inflammation and their interaction are consistent with the roles for fibrinogen and CRP found here.

In conclusion, the demonstration that both fibrinogen and CRP levels are associated with cognitive deficits suggests that repeated measures of these blood-based, compared with cerebrospinal fluid-based, biomarkers can be of some value in clinical practice. Such possibility is an ally in responding to the challenge posed by dementias in aging populations.

**Research in context**

1. Systematic review: We searched PubMed for studies on associations between higher levels of CRP and fibrinogen and lower cognitive functions. The review uncovered a lack of evidence using repeated measures of both inflammatory markers and cognitive scores. Change-scores longitudinal design, where change in scores of cognitive tests is regressed on baseline biomarker levels, has given inconsistent associations between biomarker levels and cognitive decline.
2. Interpretation: Evidence from this study supports some studies in the literature and shows higher levels of CRP and fibrinogen are related to worse episodic memory in later life, in the younger old and older old groups. But the evidence also stands in contrast to recent results which suggest that higher inflammation can be protective among the older old.
3. Future directions: Repeated measures of blood-based biomarkers (including fibrinogen, high sensitivity CRP, and interleukin 6) and multiple cognitive functions (including executive function and fluid intelligence) are needed to obtain consistent inferences. This constitutes a low hanging fruit because major longitudinal aging studies comparable with English Longitudinal Study of Aging are ongoing in the United States (Health and Retirement Study) and Europe (Survey of Health, Ageing and Retirement in Europe [SHARE]). Given the importance of dementias, repeatedly collecting blood-based biomarkers in these national samples may be clinically and scientifically fruitful.

## Figures and Tables

**Table 1:** Descriptive statistics of the analytic sample

Variable	Analytic sample, n = 14,180 person-year
Episodic memory (mean, SD)	10.5, 3.5
High sensitivity CRP, mg/L (mean, SD)	2.6, 2.3
Fibrinogen, g/L (mean, SD)	3.0, 1.0
Age, y (mean, SD)	67.3, 8.4
Sex, women (n, %)	7947, 54.4
Education, college (n, %)	4571, 31.3
Less than college degree (n, %)	10,040, 68.7
Married or cohabit (*n*, %)	10,248, 70.1
Not married or cohabit (n, %)	4363, 29.9
Wealth tertile, bottom (n, %)	4069, 27.9
Middle tertile (n, %)	5078, 34.8
Richest tertile (n, %)	5464, 37.4
Ethnicity, Caucasian (n, %)	14,360, 98.3
Ethnic minority (n, %)	251, 1.7
Occupation, prof-managerial (n, %)	5569, 38.1
Intermediate (n, %)	3469, 23.7
Routine manual (*n*, %)	5573, 38.1
Social contact, ≥ once a week (n, %)	7584, 51.9
Physical problems (mean, SD)	1.9, 2.8
Hypertensive (n, %)	6072, 41.6
Lung disease (n, %)	848, 5.8
Diabetics (n, %)	1223, 8.4
Cancer (n, %)	1214, 8.3
Heart condition (n, %)	2463, 16.9
Stroke (n, %)	487, 3.3
CES-D ≥4 (n, %)	1752, 12.0
Current smoker (n, %)	9035, 61.8
Drink regularly (n, %)	12,042, 82.4
Exercise moderately (n, %)	11,586, 79.3
Exercise vigorously (n, %)	4546, 31.1

Abbreviations: SD, standard deviation; CRP, C-reactive protein; CES-D, Center for Epidemiologic Studies Depression scale; ELSA, English Longitudinal Study of Aging.

**Table 2:** Inflammatory markers and episodic memory (coefficients and confidence intervals in parentheses) in ELSA 2004–2013

Factor	Inflammation	Age-interaction
CRP	−0.001 (−0.064 to 0.063)	0.033 (−0.036 to 0.101)
CRP and age ≥75		−0.179* (−0.320 to −0.038)
Fibrinogen	−0.149** (−0.248 to −0.050)	−0.154** (−0.254 to −0.054)
Fibrinogen and age ≥75		0.031 (−0.039 to 0.102)
N	14,180	14,180
*R*^*2*^	0.25	0.25

Abbreviations: ELSA, English Longitudinal Study of Aging; CRP, C-reactive protein.

NOTE. Adjusted for sex, age, age^2^, education, wealth, occupational class, marital status, ethnicity, social contact, physical function problems, hypertension, lung disease, diabetes, cancer, heart conditions, stroke, CES-D score, smoking, drinking, and exercise. Significant at 5% *, 1% **.
