# Supplementary material for: Repeated systemic inflammation was associated with cognitive deficits in older Britons
Source: Alzheimers Dement (Amst). 2015 Dec 28;3:1–6. doi: 10.1016/j.dadm.2015.11.009 (PMC4879642; doi:10.1016/j.dadm.2015.11.009)
Supplement: Supplementary Material [file mmc1.docx]

**Appendix**

Table 3. Inflammatory markers and episodic memory (coefficients and confidence intervals in square brackets) in ELSA 2004-2013

|  | Inflammation | | Plus old age interaction | |
| --- | --- | --- | --- | --- |
|  | Coeff. | 95% CI | Coeff. | 95% CI |
| Age | 0.403^***^ | [0.313,0.492] | 0.399^***^ | [0.299,0.500] |
| Age^2^ | -0.004^***^ | [-0.005,-0.003] | -0.004^***^ | [-0.005,-0.003] |
| Sex, Female | 0.920^***^ | [0.790,1.050] | 0.920^***^ | [0.790,1.049] |
| Occupation, intermediate | 0.754^***^ | [0.593,0.916] | 0.755^***^ | [0.593,0.916] |
| Managerial | 1.062^***^ | [0.911,1.213] | 1.061^***^ | [0.911,1.212] |
| Marital, married/cohab | -0.085 | [-0.216,0.046] | -0.086 | [-0.217,0.045] |
| Some college | 0.789^***^ | [0.642,0.936] | 0.790^***^ | [0.643,0.937] |
| Wealth, middle tertile | 0.173^**^ | [0.044,0.303] | 0.175^**^ | [0.046,0.305] |
| Top tertile | 0.393^***^ | [0.251,0.536] | 0.396^***^ | [0.254,0.538] |
| Ethnicity, minority | -1.137^***^ | [-1.573,-0.702] | -1.133^***^ | [-1.569,-0.698] |
| Physical function problems | -0.041^***^ | [-0.063,-0.020] | -0.041^***^ | [-0.063,-0.020] |
| Hypertensive | -0.016 | [-0.133,0.101] | -0.016 | [-0.134,0.101] |
| Lung problems | 0.087 | [-0.152,0.326] | 0.086 | [-0.153,0.325] |
| Diabetics | -0.123 | [-0.323,0.077] | -0.124 | [-0.324,0.075] |
| Cancer | 0.223^*^ | [0.025,0.422] | 0.222^*^ | [0.023,0.420] |
| CVD | 0.237^**^ | [0.087,0.388] | 0.238^**^ | [0.088,0.389] |
| Stroke | -0.196 | [-0.503,0.111] | -0.201 | [-0.508,0.106] |
| Current smoker | -0.074 | [-0.195,0.048] | -0.075 | [-0.196,0.047] |
| Drink daily | 0.693^***^ | [0.555,0.831] | 0.695^***^ | [0.557,0.833] |
| Exercise moderately | 0.249^***^ | [0.117,0.380] | 0.247^***^ | [0.115,0.378] |
| Exercise vigorously | 0.141^*^ | [0.029,0.252] | 0.143^*^ | [0.032,0.255] |
| CESD | -0.076^***^ | [-0.106,-0.046] | -0.076^***^ | [-0.106,-0.046] |
| Log hsCRP | -0.001 | [-0.064,0.063] | 0.033 | [-0.036,0.101] |
| Fibrinogen | -0.149^**^ | [-0.248,-0.050] | -0.154^**^ | [-0.254,-0.054] |
| Log hsCRP & age≥75 |  |  | -0.179^*^ | [-0.320,-0.038] |
| Fibrinogen & age≥75 |  |  | 0.031 | [-0.039,0.102] |
| *N* | 14180  0.25 | | 14180  0.25 | |
| *R^2^* |  |  |  |  |

95% confidence intervals in brackets

^*^ *p* < 0.05, ^**^ *p* < 0.01, ^***^ *p* < 0.001
